# Reconstruction of the temporomandibular joint using a vascularized medial femoral condyle osteocartilaginous flap: an experimental investigation in miniature pigs

**DOI:** 10.1186/s12903-023-03341-z

**Published:** 2023-09-01

**Authors:** Tianyi Lin, Shuo Chen, Long Xia, Bimeng Jie, Yi Zhang, Yang He

**Affiliations:** 1 Department of Oral and Maxillofacial Surgery, Peking University School and Hospital of Stomatology, No.22 Zhongguancun South Avenue, Haidian District, Beijing, 100081 PR China; 2 National Center for Stomatology, Beijing, PR China; 3 National Clinical Research Center for Oral Diseases, Beijing, PR China; 4 National Engineering Research Center of Oral Biomaterials and Digital Medical Devices, Beijing, PR China; 5 Beijing Key Laboratory of Digital Stomatology, Beijing, PR China

**Keywords:** Temporomandibular joint, Mandibular reconstruction, Medial femoral condyle flap, Pig

## Abstract

**Background:**

Reconstruction of the temporomandibular joint (TMJ) is a significant challenge in maxillofacial surgery. A vascularized medial femoral condyle (MFC) osteocartilaginous flap is a good choice for TMJ reconstruction. In this study, we evaluated the radiographic and histological changes of MFC after TMJ reconstruction.

**Methods:**

A ramus-condyle unit (RCU) defect was created unilaterally in five adult male Bama miniature pigs. The ipsilateral vascularized MFC osteocartilaginous flap was used to reconstruct the TMJ, and the non-operative sides served as controls. Multislice spiral computed tomography (CT) was performed preoperatively, immediately postoperatively, and at two weeks, three months, and six months postoperatively. Three animals were euthanized at 6 months postoperatively. Their reconstructed condyles, natural condyles and the MFCs on the opposite side were collected and subjected to µCT and histological evaluation.

**Results:**

In the miniature pigs, the vascularized MFC osteocartilaginous flap was fused to the mandible, thus restoring the structure and function of the RCU. The postoperative radiographic changes and histological results showed that the reconstructed condyle was remodeled toward the natural condyle, forming a similar structure, which was significantly different from the MFC.

**Conclusions:**

In miniature pigs, the RCU can be successfully reconstructed by vascularized osteocartilaginous MFC flap. The reconstructed condyle had almost the same appearance and histological characteristics as the natural condyle.

**Supplementary Information:**

The online version contains supplementary material available at 10.1186/s12903-023-03341-z.

## Background

The temporomandibular joint (TMJ) has a number of important functions, such as mastication and speech. The articulatory system comprises the condyles, intra-articular discs, mandibular muscles and the occlusion. Due to the ‘knock-on effect’, abnormalities in any side or in any single part of the system can exert an overall impact on the joint [1]. Malformations [2, 3], tumors [4, 5], and ankylosis [6] are among the conditions that may cause a ramus-condyle unit (RCU) defect, which requires reconstruction [7]. The complex anatomic and physiologic features of TMJ make its reconstruction challenging. Chondro-costal graft (CCG), vascularized autogenous tissue transfer, and alloplastic TMJ replacement have been applied for TMJ reconstruction [8].

Although various reconstruction methods have been implemented in the past, each of them has had its limitations. Hence, new alternative techniques need to be explored and established. In this respect, in 2014, Thiele et al. proposed that vascularized medial femoral condyle (MFC) flaps can supply the bone, cartilage, and skin with minimal donor site morbidity and have the potential for TMJ reconstruction [9]. In the same year, Lee et al. reported a successful TMJ reconstruction using the femoral medial epicondyle free flap to treat malunion of a subcondylar fracture without the use of articular cartilage [10]. Further, in 2015, Wong et al. introduced the application of a vascularized lateral femoral condyle (LFC) flap, a new flap similar to the MFC flap, in hand surgery [11]. Subsequently, Enzinger et al. used a vascularized LFC osteocartilaginous flap to repair mandibular condyle defects with good functional and morphological outcomes [12]. Since 2019, we have reconstructed TMJs using vascularized MFC osteocartilaginous flaps in TMJ ankylosis and tumor patients. Our three-year follow-up data indicate that satisfactory treatment results have been achieved [13].

The mandible and long bones have different developmental mechanisms, structures and functions. The surface of the medial femoral condyle is covered with hyaline cartilage, which is compatible with the function of the knee joint, having cushioning and friction-reducing functions [14]. Unlike most joints, the condyle surface is covered by fibrocartilage rather than hyaline cartilage [15]. Condylar cartilage plays an important role in mandible development and is adapted to the motional characteristics of the TMJ [1, 16, 17]. However, it is unclear whether post-transplantation MFC is structurally modified to accommodate the new function.

Among the large animals used for TMJ reconstruction research, pigs are the most suitable, since their joint, disc morphology and mandibular movement patterns are similar to those of humans [18]. Moreover, the vascularized MFC flap has only been studied in miniature pigs [19, 20]. In the present study, we developed a miniature pig model of a unilateral vascularized MFC osteocartilaginous flap to regenerate an RCU defect. Then we studied the postoperative remodeling of the reconstructed condyle.

## Methods

### Animals

Five adult male Bama miniature pigs, aged 12–18 months and weighing 30–40 kg, were used in this experiment. All experiments were approved by the Biomedical Ethical Committee of Peking University (No. LA2022367). The experimental animals were normal grade, produced by Tianjin Binong Experimental Animal Breeding Technology Co., Ltd. They were raised and operated on in a barrier environment in Beijing Tonghe Litai Biotechnology Co., Ltd. The experimental animals were acclimated and observed for two weeks before the operation to ensure their health. The experimental animals were raised in iron cages with sufficient space to move around, supplemented with water and feed by specially assigned staff. All animals were treated as the experimental group with the nonoperative side as the control. The animals were euthanized by excessive anesthesia after six months. The data of animals with serious adverse events, such as accidental death, condylar fracture, necrosis, and infection, were excluded (n ≥ 3 after exclusion) during the analysis of the results.

### Surgical protocols

**Fig. 1:**
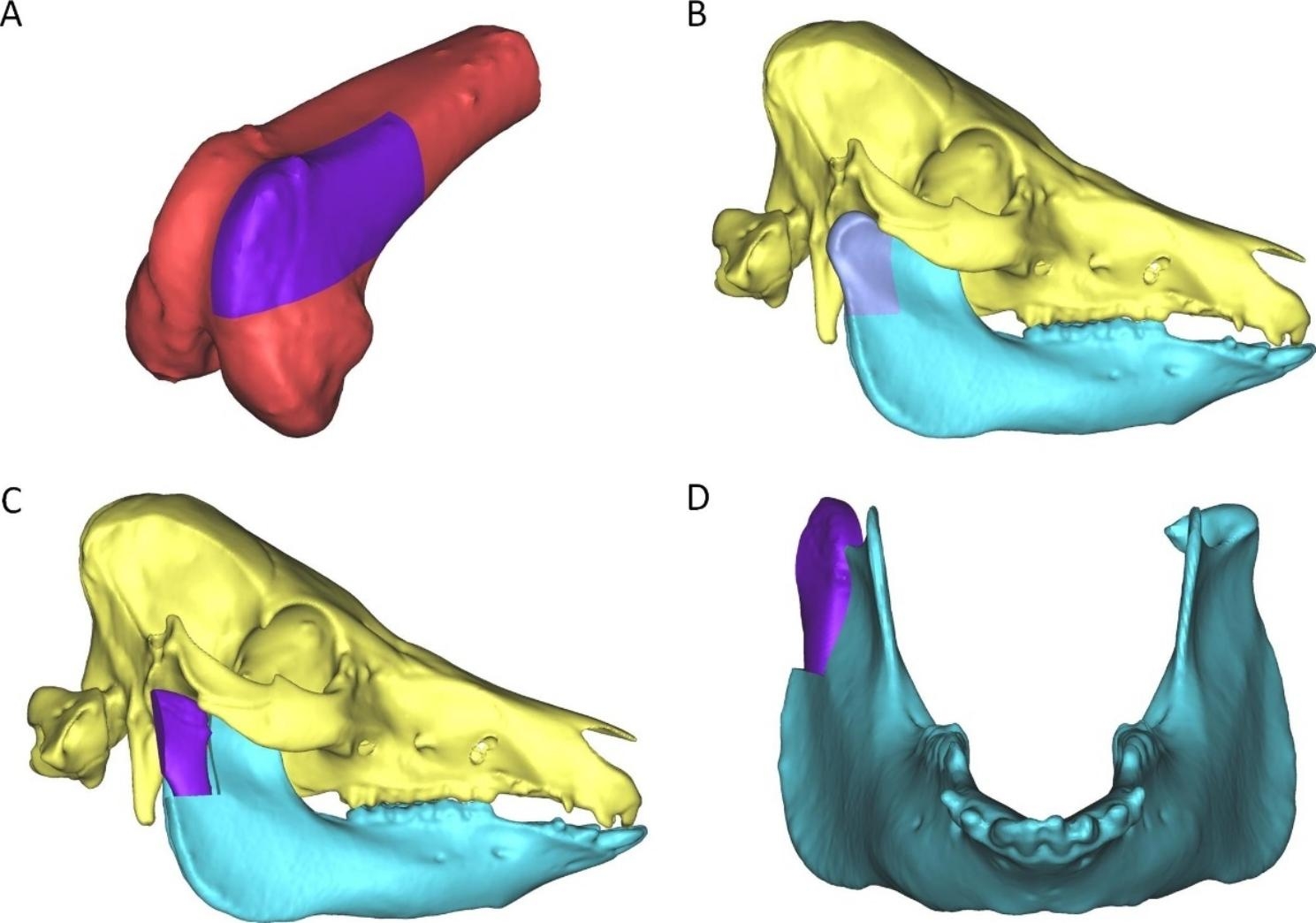
Diagrams of RCU reconstruction by vascularized MFC osteocartilaginous flap. **A** Range of the vascularized MFC osteocartilaginous flap. **B** Defect range of RCU. **C** and **D** TMJ reconstruction result

All operations were performed by one surgical team. Dr. He completed the core operation steps, and other authors participated in the operations as assistants. The surgical team has completed more than 10 cases of TMJ reconstruction using MFC flap in clinic. The animals were fasted for 12 h before surgery preoperatively, and anesthesia was induced by intramuscular injection of xylazine hydrochloride (40 mg/kg) and Zoletil 50 (zolazepam + tiletamine) (5 mg/kg). Preoperative multislice spiral computed tomography (CT) was performed after preparing the skin of the right face and hind limb. Then, an MFC osteocartilaginous flap and RUC reconstruction were designed using CT data (Fig. 1). Next, the experimental animal was placed on the operating table, with the upper body in the right lateral position and the lower body in the supine position. After tracheal intubation, the respiratory anesthesia machine was connected, and anesthesia was maintained with isoflurane inhalation throughout the operation.

**Fig. 2:**
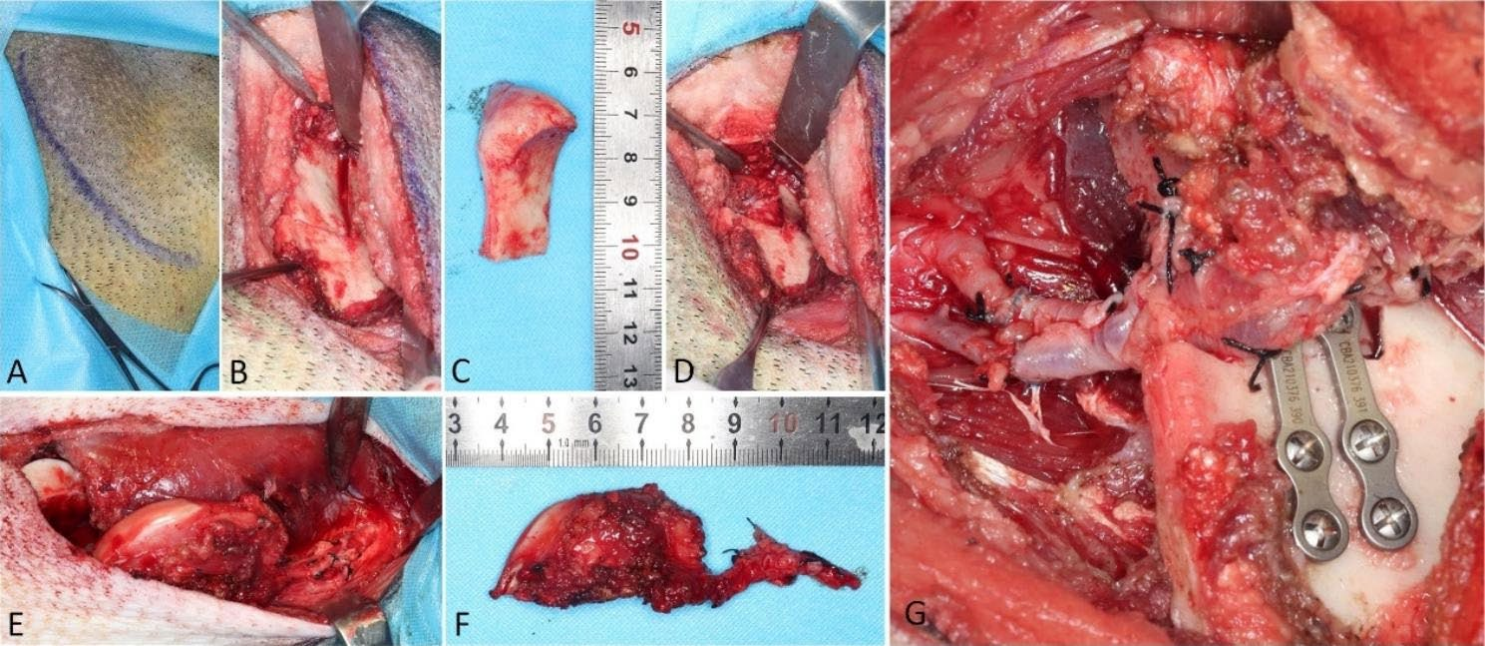
Surgical procedure. **A** Incision marked for the retromandibular approach. **B** The mandibular ramus and condyle were exposed, and osteotomy was performed. **C** The removed RCU. **D** RCU defect. **E-F** Collection of the MFC osteocartilaginous flap. **G** Vascular anastomosis and bone flap fixation

After the animal was fixed, intravenous access was obtained from the dorsal auricular vein. Penicillin and ibuprofen were injected before the operation, and 1000 mL of glucose and sodium chloride were infused intravenously during the operation. The operation area was disinfected with povidone-iodine, and sterile sheets were placed. The incision of the retromandibular approach was marked (Fig. 2A), and the skin and subcutaneous tissue were incised. The mandibular angle and the posterior margin of the ramus were exposed, and the attachment of the masseter muscle was cut off; the lateral surface of the ramus and condyle were then exposed (Fig. 2B). The ramus was sawed downward along the anterior margin of the condyle, at an approximate width of the bone of 2 cm. A horizontal osteotomy was performed at approximately 4 cm from the apex of the condyle; the RCU was removed, but the articular disc was retained (Fig. 2C, D). The maxillary artery and its accompanying vein located medial to the ramus were dissected and reserved.

A 10-cm incision was made on the medial side of the right hind limb above the knee. The medial femoris muscle was stripped forward to expose the medial femur, the descending genicular artery and its accompanying vein were dissected, and then part of the femoral artery and vein were separated and carried toward the proximal end. The approximate pedicle length was 5 cm. An osteocartilaginous flap with a length of 4 cm, a width of 2 cm and a thickness of 0.8 cm was cut off and transferred to the facial surgery area (Fig. 2E-F). The leg wound was sutured layer-by-layer.

The femoral artery and vein of the osteocartilaginous flap were anastomosed end-to-end with the maxillary artery and vein, and the blood supply and reflux were checked. The osteocartilaginous flap was fixed with two CIBEI (Ningbo, China) 4-hole short-bridge mini titanium plates and eight 8-mm screws (Fig. 2G). The wound was sutured layer-by-layer.

Isoflurane was stopped, and the endotracheal tubes were extubated after spontaneous breathing recovered. The animals were returned to the cage after postoperative spiral CT examination. After the operation, penicillin and ibuprofen were injected intramuscularly for 14 days, and body temperature was monitored for five days. For the first week after the operation, the pigs were given a semi-liquid diet: the feed was broken up and soaked into a paste. A normal solid diet was resumed one week afterwards.

### Multi-slice spiral CT

To examine the results of TMJ reconstruction and observe postoperative condylar remodeling, all the animals were subjected to five CT examinations: preoperatively, immediately postoperatively, and at two weeks, three months, and six months postoperatively. The examination procedure performed preoperatively and immediately postoperatively has been described above. Anesthesia induction via intramuscular injection was performed at other time points before CT examination. A 64-row, 128-slice CT scanner (United Imaging uCT 760; Shanghai, China) was employed for the examinations at 238 mA and 120 kV, and a layer thickness of 0.625 mm.

### µCT examination

After the animals were euthanized, the entire mandibles were isolated, and the left MFCs were cut within the MFC flap range. After the samples were photographed, the reconstructed RCUs and the nonoperative RCUs were cut according to the osteotomy range of the intraoperative RCU defect and fixed with 10% formalin along with the MFCs. µCT examinations of the samples were conducted by Xi’an Aoyun Electronic Technology Co., Ltd. with µCT, the equipment was Always Imaging AX2000, at 15-µm resolution. Data of the following parameters were collected and analyzed in this study: bone volume to total volume (BV/TV), bone surface to bone volume (BS/BV), trabecular thickness (Tb.Th.), trabecular number (Tb.N.), and trabecular spacing (Tb.Sp.).

### Histologic examination

The samples subjected to µCT examination were dissected by the dynamic system along the sagittal plane in the middle of the condyle and then decalcified with EDTA decalcified solution for three months. After full decalcification, five-micron-thick sections were prepared after the samples were embedded in paraffin. Then, the slides were subjected to H&E and Safranin O-fast green stanning using standard protocols, followed by photographing under a microscope.

### Statistical analysis

All quantitative data are expressed as mean ± S.D. Friedman test and Dunn’s multiple comparisons test were employed for comparison among groups. A P-value < 0.05 was considered to indicate a statistically significant difference. Statistical analysis was performed using GraphPad Prism 9.0.

## Results

### Animal health

All the animals were alive with a normal body temperature five days after the operation (Additional Fig. 1)., The normal dry weight food intake was resumed from 3 to 5 days postoperatively. The pigs recovered to normal activities 1–2 weeks postoperatively. CT examinations revealed that the reconstructed condyle in one pig had split at two weeks. In another case, the operative area had been infected and failed to recover after debridement. These aforementioned two pigs were removed from the study due to complications that could have affected the experimental results. The three remaining pigs survived to 6 months postoperatively, when each of them weighed approximately 40 kg (Additional Fig. 2).

### Multi-slice spiral CT images

**Fig. 3:**
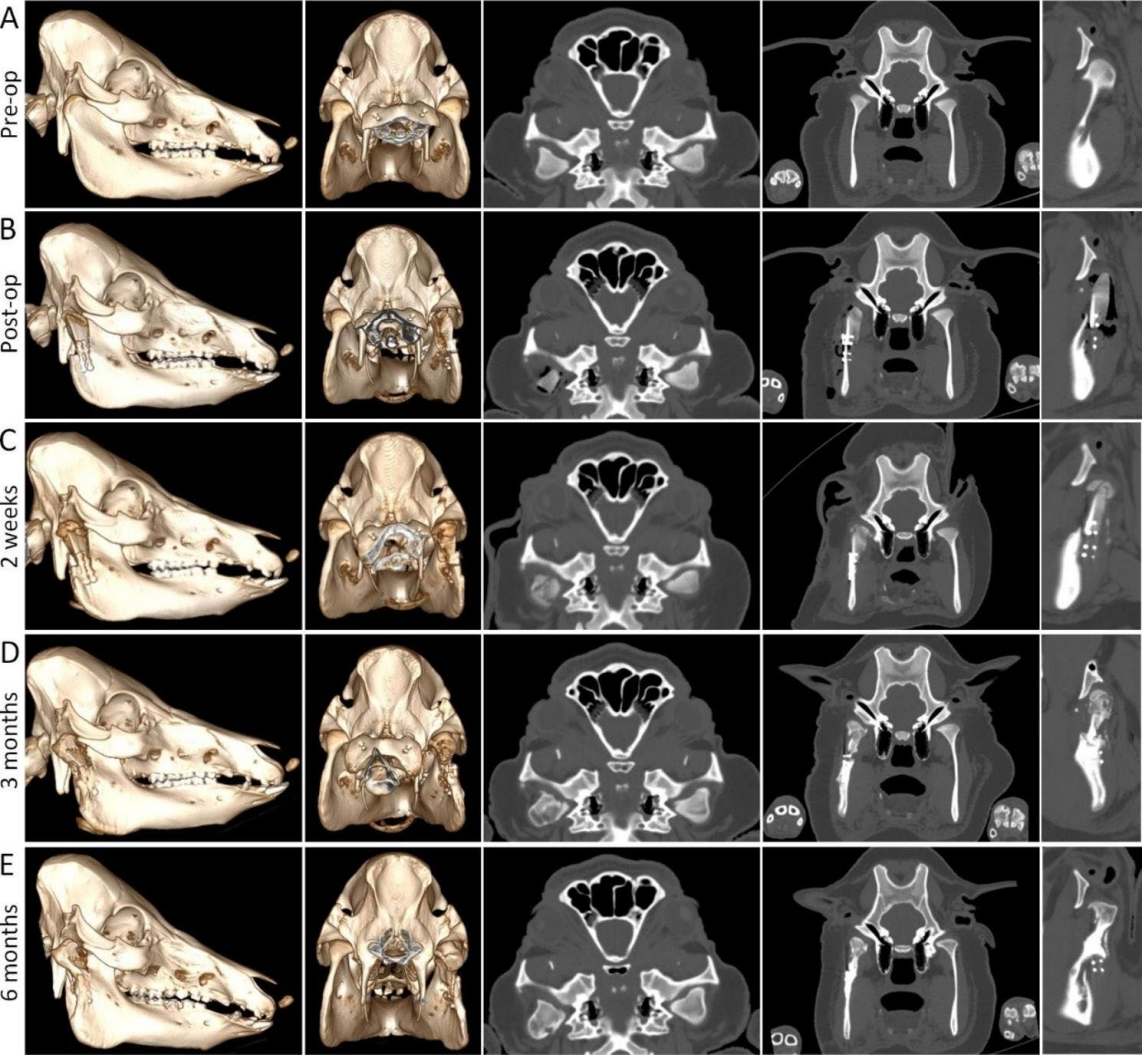
CT imaging at different stages. CT images acquired before (**A**), immediately after the operation (**B**), and at 2 weeks (**C**), 3 months (**D**) and 6 months (**E**) after the operation

The comparison between the preoperative (Fig. 3A) and immediately postoperative (Fig. 3B) CT images showed that a symmetrical shape of the mandible and a well-recovered ramus height had been achieved. Bone formation around the reconstructed condyle was observed at two weeks after the operation (Fig. 3C). At three months postoperatively, the reconstructed condyle was obviously remodeled, with a natural condyle appearance. The shape and width of the two condyles were very similar, but the bone density was still uneven (Fig. 3D). At six months after the operation, the shape of the reconstructed condyle had no significant change when compared with that at three months, but the bone density of the reconstructed condyle was more uniform, which was close to that of the natural condyle (Fig. 3E).

### Sample observation

**Fig. 4:**
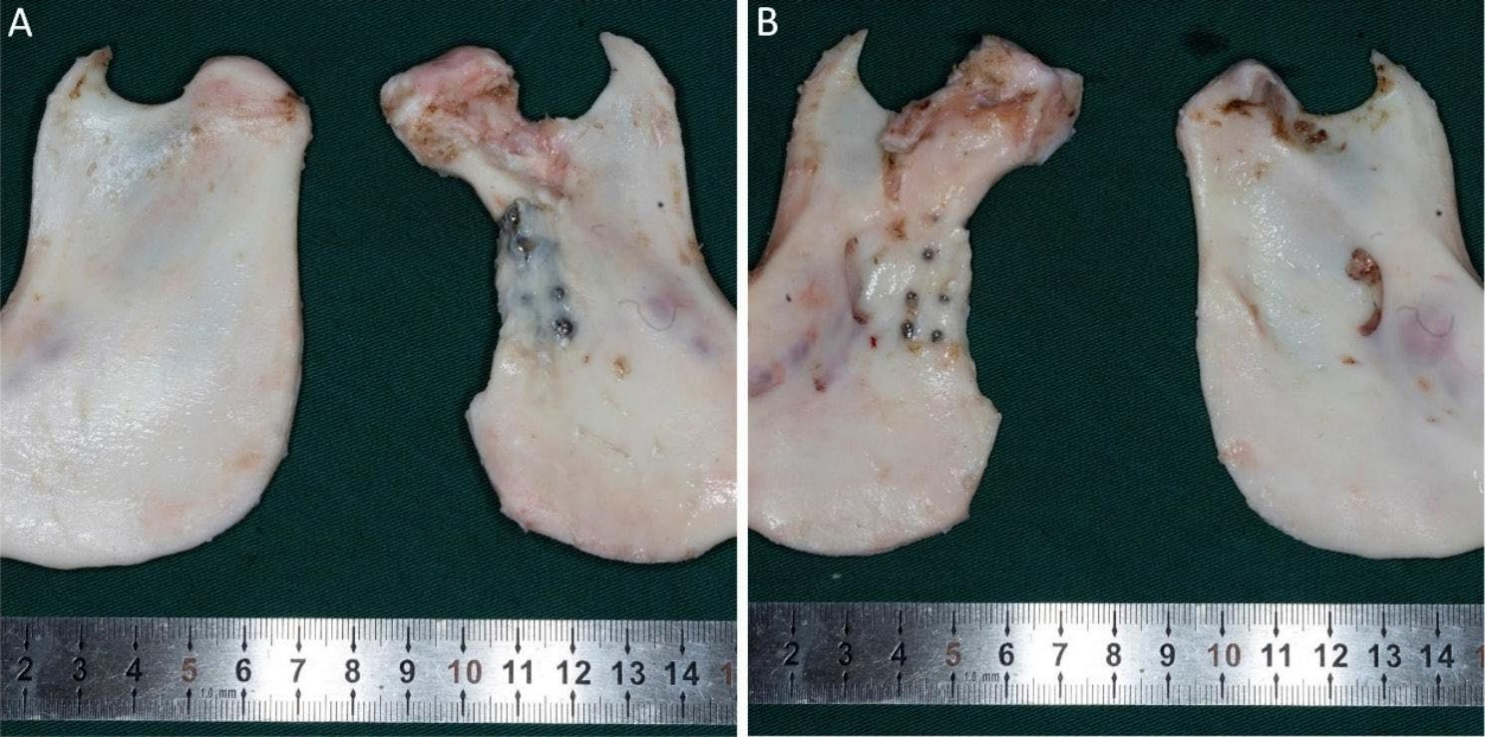
Mandibular samples. **A** Lateral view of the mandibular ramus. **B** Medial view of the mandibular ramus

Visual observation showed that the bone flap had fully fused with the ramus of the mandible without an obvious boundary; some titanium plates and screws were covered by the newly formed bone. The shape and texture of the reconstructed condyle were similar to those of the natural condyle. The articular surface of the reconstructed condyle was smooth and light red, which was significantly different from the pure white articular surface of the MFC. The unstressed area of the reconstructed condyle retained some articular cartilage without obvious remodeling (Fig. 4).

### µCT images

**Fig. 5:**
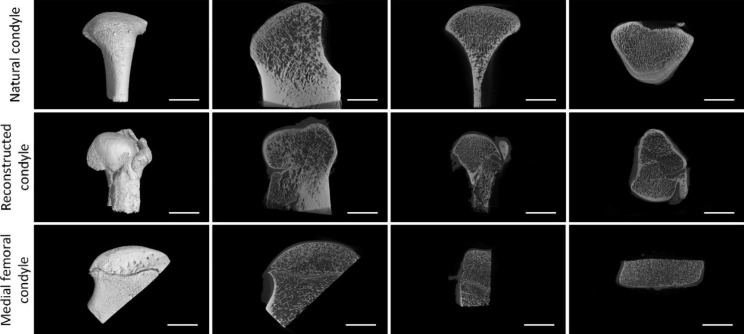
µCT examination results.µCT images of the natural condyle, reconstructed condyle and MFC. Scale bars, 10 mm

The morphology and trabecular distribution of the reconstructed condyle were consistent with those of the natural condyle but were significantly different from those of the MFC (Fig. 5). The results of the quantitative analysis supported this basic impression. There were significant differences in BV/TV, BS/BV and Tb.Th. between the reconstructed condyles and the MFC, but no significant difference in Tb.N. and Tb.Sp was found. The reconstructed condyles had larger BV/TV and Tb.Th., but lower BS/BV values. (Table 1).

**Table 1:** µCT quantification of the subchondral bone

	Mean ± S.D.			Friedman test		Dunn’s multiple comparisons test					
						NC vs. RC		NC vs. MFC		RC vs. MFC	
	NC	RC	MFC	P-value	Summary	P-value	Summary	P-value	Summary	P-value	Summary
BV/TV (%)	0.4254 ± 0.06317	0.4759 ± 0.06980	0.3174 ± 0.02780	0.0278	*	0.662	ns	0.662	ns	0.0429	*
BS/BV (1/mm)	14.28 ± 1.276	12.31 ± 1.069	17.36 ± 0.3773	0.0278	*	0.662	ns	0.662	ns	0.0429	*
Tb.Th. (mm)	0.1411 ± 0.01171	0.1656 ± 0.01575	0.1167 ± 0.005774	0.0278	*	0.662	ns	0.662	ns	0.0429	*
Tb.N. (1/mm)	3.008 ± 0.1840	2.843 ± 0.1571	2.756 ± 0.2480	0.1944	ns	0.3074	ns	0.1237	ns	> 0.9999	ns
Tb.Sp. (mm)	0.1967 ± 0.03180	0.1867 ± 0.03215	0.2467 ± 0.03215	0.1944	ns	> 0.9999	ns	0.3074	ns	0.1237	ns

Bone volume to total volume (BV/TV), bone surface to bone volume (BS/BV), trabecular thickness (Tb.Th.), trabecular number (Tb.N.), and trabecular spacing (Tb.Sp.) of the natural condyle (NC), reconstructed condyle (RC) and medial femoral condyle (MFC).

*P-value < 0.05

### Histologic analysis

**Fig. 6:**
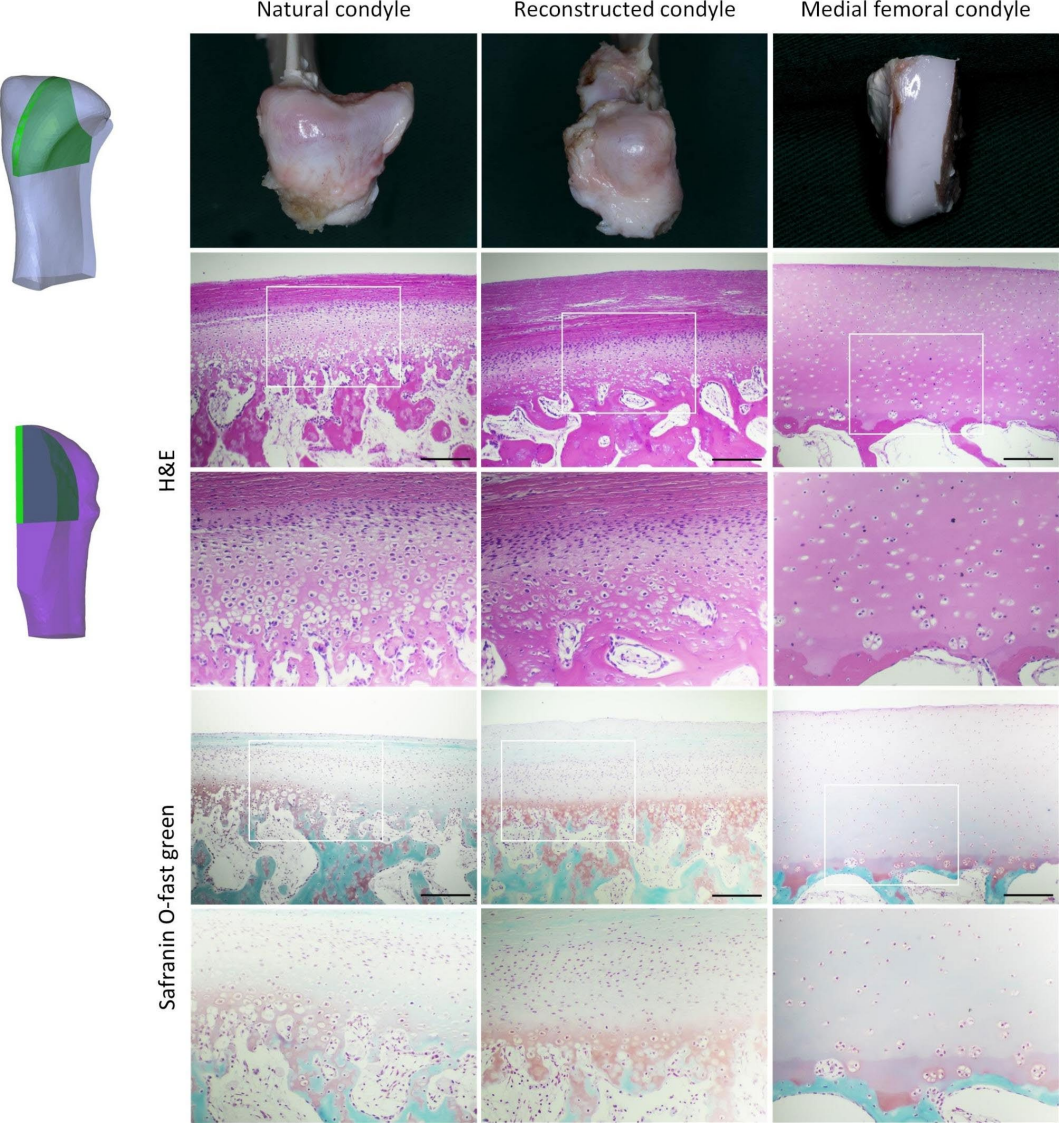
Schematic and representative photographs and H&E, Safranin O-fast green staining of the natural condyle, reconstructed condyle and MFC from the sagittal condylar cross sections. The white box indicates the range below. Scale bar, 100 μm

The articular cartilage of the reconstructed condyle was similar to that of the natural condyles, and could be divided into the following zones: fibrous articular surface zone, cellular rich zone, fibrocartilaginous zone, and cartilage calcified zone. While the articular cartilage of MFC was not significantly stratified, no fibrous tissue was observed on the cartilage surface. In addition, the cartilage layers of the reconstructed condyle and the natural condyle were significantly thinner than that of the MFC. Similar to the µCT findings, the bone trabeculae of the MFC were significantly thinner and sparser, with a lower bone density (Fig. 6).

## Discussion

Although there are several methods for TMJ reconstruction, it is still a great challenge for maxillofacial surgeons [7, 21–23]. In this study, we evaluated the radiographic and histological changes of MFC osteocartilaginous flap after graft to TMJ in miniature pigs and obtained satisfactory results.

In 1991, Sakai et al. reported the utility of a vascularized MFC flap that was supplied by the descending genicular artery, containing periosteum and thin cortical bone, for the treatment of nonunion after fracture of the ulna, humerus, and metacarpals; the skin was also taken in one case [24]. The vascularized MFC flap is multifunctional and provides bone, cartilage, periosteum, fascia, muscle, tendon, and skin. This technique is commonly applied for the repair of complex defects of the extremities [25–27]. In 2020, Deng et al. reported a series of cases of patients treated with vascularized MFC flaps for small bone defects and post-fracture nonunion in three centers in three countries. The recipient areas included the scaphoid, lunate, metacarpal, tibia, ulna, navicular bone, and clavicle [28].

In the head and neck, the MFC flap was used for the reconstruction of orbital, alveolar, maxillary, mandibular, skull, and laryngotracheal scaffolds, but none contained cartilage [29, 30]. In 2014, Thiele et al. proposed that the vascularized MFC flap could provide articular cartilage and could be used for the repair of TMJ defects [9]. In the same year, Lee et al. reported a TMJ reconstruction with a vascularized MFC flap but without articular cartilage [10]. In 2021, we published our results from the use of vascularized MFC osteocartilaginous flaps for TMJ reconstruction in a series of patients with TMJ ankylosis and tumors, which achieved good therapeutic results [13].

To date, animal experimental studies on the MFC flap have been performed only in pigs. In 2016, Borumandi et al. studied the long-term impact of arterialized venous bone flaps employed in pig MFC [19]. In 2018, Higgins et al. compared the long-term cartilage quality of vascularized versus nonvascularized osteocartilaginous flaps of the MFC [20]. In these two studies, the MFC flap was left in situ without transfer, but the feasibility of harvesting vascularized MFC flaps in pigs was reported. To the best of our knowledge, there is no animal experimental study in which the MFC flap was transferred to the recipient site. Therefore, the present study is the first animal experimental investigation of the use of vascularized MFC osteocartilaginous flaps in TMJ reconstruction.

Alternative techniques and grafts, including CCG and distraction osteogenesis, have been explored for TMJ reconstruction in animals. A study on the reconstruction of the TMJ by CCG in growing pigs showed no significant difference in the growth of the ribs between the joint area and the non-stressed area; only growth plate compression was established [31]. A rhesus monkey study was conducted, in which the condyle was reconstructed via distraction osteogenesis (with disc removal). At 24 weeks after the operation, the surface of the reconstructed condyle was covered by a thick fibrous cap, and a thick cartilage layer was formed below it, which was also different from that of the natural condyle [32]. Later, research attention has been focused on the importance of articular cartilage regeneration through tissue engineering. In 2020, Chen et al. used tissue engineering to implant osteocytes and chondrocytes in layers on a bovine bone RCU scaffold and created an appearance of the reconstructed condyles that was close to that of the natural condyle in miniature pigs [33]. The reconstruction of the condyle using MFC osteocartilaginous flap had the closest histological resemblance to that of the natural condyle as compared to ones achieved in previous studies, and the histological structure was highly ordered. The remodeling of this structure may allow for better adaptation to the TMJ function.

The bone regeneration rate of the minipig mandible (1.2–1.5 μm/day) has been reported to be comparable to that of humans (1.0–1.5 μm/day) [34].However, in clinical cases, of middle-aged and elderly patients mainly, the reconstructed condyles rarely turned into a shape similar to that of the natural condyle, and only a certain degree of bone density change can be observed [13]. Therefore, the results of animal experiments are only of certain comparative and reference value, and the actual clinical effect should not be overestimated. The experimental results in obtained miniature pigs suggest that vascularized MFC flap can have high clinical applications opportunities after validation in further clinical trials.

CCG and TMJ prosthesis are also widely accepted methods of joint reconstruction [22, 23]. CCG has the advantage of preserving growth potential in young patients, especially suitable for hemifacial microsomia [2]. Total TMJ prosthesis is the mainstream temporomandibular joint reconstruction method and research direction for adults, with good therapeutic effect and wide application [39]. We defined the medial femoral condylar flap as an alternative technique, although it is not optimal in many cases and not suitable for children and adolescents. Given that commercial TMJ prostheses are rare, expensive and not readily available in many hospitals in developing countries, vascularized flaps can be alternative methods. In addition, vascularized flaps can carry a variety of tissues and withstand radiotherapy, which has unique advantages in tumor patients.

Maintenance or reconstruction of the articular disc is considered a part of TMJ reconstruction. However, patients requiring condylar reconstruction often do not have an available articular disc (ankylosis) or cannot retain the disc (tumors) [35, 36]. In clinical practice, we generally use temporalis myofascial flap and abdominal fat graft to replace the buffering function of the articular disc [37, 38]. Relevant researches on artificial TMJ disc have achieved satisfactory results in animal models [40–42]. However, no clinical study has been performed, which is one of the research directions in the future.

## Conclusions

The MFC reconstructed condyle had almost the same appearance and histological characteristics as the natural condyle in miniature pigs. Therefore, MFC has the potential to serve as an alternative method for TMJ reconstruction.

### Electronic supplementary material

Below is the link to the electronic supplementary material.

Supplementary Material 1

## Data Availability

The data sets used and/or analyzed during the current study are available from the corresponding author upon reasonable request.

## Abbreviations

BS/BV: Bone surface/bone volume ratio
BV/TV: Bone volume/total volume ratio
CCG: Costochondral grafts
CT: Computed tomography
LMC: Lateral femoral condyle
MFC: Medial femoral condyle
RCU: Ramus-condyle unit
Tb. N.: Trabecular number
Tb. Sp.: Trabecular separation
Tb. Th.: Trabecular thickness
TMJ: Temporomandibular joint
