# Supplementary material for: Reconstruction of the temporomandibular joint using a vascularized medial femoral condyle osteocartilaginous flap: an experimental investigation in miniature pigs
Source: BMC Oral Health. 2023 Sep 1;23:621. doi: 10.1186/s12903-023-03341-z (PMC10474687; doi:10.1186/s12903-023-03341-z)
Supplement: Supplementary file 1 — Supplementary Material 1 [file 12903_2023_3341_MOESM1_ESM.docx]

**Supplementary Information**

**Additional Table 1** ARRIVE Essential 10.

| ARRIVE Essential 10 | | |
| --- | --- | --- |
| Study design | 1 | For each experiment, provide brief details of study design including: a. The groups being compared, including control groups. If no control group has been used, the rationale should be stated. b. The experimental unit (e.g., a single animal, litter, or cage of animals). |
| Sample size | 2 | a. Specify the exact number of experimental units allocated to each group, and the total number in each experiment. Also indicate the total number of animals used. b. Explain how the sample size was decided. Provide details of any a priori sample size calculation, if done. |
| Inclusion and exclusion criteria | 3 | a. Describe any criteria used for including and excluding animals (or experimental units) during the experiment, and data points during the analysis. Specify if these criteria were established a priori. If no criteria were set, state this explicitly. b. For each experimental group, report any animals, experimental units, or data points not included in the analysis and explain why. If there were no exclusions, state so. c. For each analysis, report the exact value of n in each experimental group. |
| Randomisation | 4 | a. State whether randomisation was used to allocate experimental units to control and treatment groups. If done, provide the method used to generate the randomisation sequence. b. Describe the strategy used to minimise potential confounders such as the order of treatments and measurements, or animal/cage location. If confounders were not controlled, state this explicitly. |
| Blinding | 5 | Describe who was aware of the group allocation at the different stages of the experiment (during the allocation, the conduct of the experiment, the outcome assessment, and the data analysis). |
| Outcome measures | 6 | a. Clearly define all outcome measures assessed (e.g., cell death, molecular markers, or behavioural changes). b. For hypothesis-testing studies, specify the primary outcome measure, i.e., the outcome measure that was used to determine the sample size. |
| Statistical methods | 7 | a. Provide details of the statistical methods used for each analysis, including software used. b. Describe any methods used to assess whether the data met the assumptions of the statistical approach, and what was done if the assumptions were not met. |
| Experimental animals | 8 | a. Provide species-appropriate details of the animals used, including species, strain and substrain, sex, age or developmental stage, and, if relevant, weight. b. Provide further relevant information on the provenance of animals, health/immune status, genetic modification status, genotype, and any previous procedures. |
| Experimental procedures | 9 | For each experimental group, including controls, describe the procedures in enough detail to allow others to replicate them, including: a. What was done, how it was done, and what was used. b. When and how often. c. Where (including detail of any acclimatisation periods). d. Why (provide rationale for procedures). |
| Results | 10 | For each experiment conducted, including independent replications, report: a. Summary/descriptive statistics for each experimental group, with a measure of variability where applicable (e.g., mean and SD, or median and range). b. If applicable, the effect size with a confidence interval. |

**1.** A ramus-condyle unit (RCU) defect was created unilaterally in adult miniature pigs. The ipsilateral vascularized medial femoral condyle (MFC) osteocartilaginous flap was used to reconstruct defect. **a.** All animals were treated as the experimental group with the nonoperative side as the control. Referring to relevant studies mentioned in discussion section, control groups are not necessary. **b.** The experimental unit is a single animal.

**2. a.** We used a total number of five experimental animals. **b.** This was an exploratory study, and in consideration of ethical, cost, surgical risk, and statistical analysis requirements, we added two animals to the minimum three to ensure that valuable and reliable experimental results could be obtained.

**3.** **a.** Adult male miniature pigs were used in this experiment. The experimental animals were acclimated and observed for two weeks before the operation to ensure their health. When analyzing the results, experimental animals with serious adverse events such as accidental death, condylar fracture, necrosis, and infection were excluded. These criteria were established a priori. **b.** CT examinations revealed that the reconstructed condyle in one pig had split at two weeks. In another case, the operative area had been infected and failed to recover after debridement. These aforementioned two pigs were removed from the study due to complications that could have affected the experimental results. **c.** The exact value of n in each analysis was 3.

**4.** Due to the small sample size and the lack of a control group, the randomization method was not implemented in this study.

**5.** No blind method was used in this study

**6. a.** The natural condyle was used as the standard for qualitative and quantitative comparison. **b.** The primary outcome was histological features, not quantitative data.

**7.** Friedman test and Dunn's multiple comparisons test were employed for comparison among groups. A P-value<0.05 was considered to indicate a statistically significant difference. Statistical analysis was performed using GraphPad Prism 9.0.

**8. a.** Adult male Bama miniature pigs, aged 12-18 months and weighing 30-40 kg, were used in this experiment. **b.** The experimental animals were normal grade wild type, produced by Tianjin Binong Experimental Animal Breeding Technology Co., Ltd.

**9.** **a. & b.** In the methods section, we described the perioperative details and the time point of examination. **c.** Surgery and CT examinations are performed at Beijing Tonghe Litai Biotechnology Co., Ltd. The experimental animals were acclimated and observed for two weeks before the operation to ensure their health. **d.** We consider that researchers in the same field can understand the reasons for all operations.

**10. a.** We provided summary/descriptive statistics in the results section. **b.** The confidence interval is 95%.

**Additional Table 2** ARRIVE Recommended Set.

| Recommended Set | | |
| --- | --- | --- |
| Abstract | 11 | Provide an accurate summary of the research objectives, animal species, strain and sex, key methods, principal findings, and study conclusions. |
| Background | 12 | a. Include sufficient scientific background to understand the rationale and context for the study, and explain the experimental approach. b. Explain how the animal species and model used address the scientific objectives and, where appropriate, the relevance to human biology. |
| Objectives | 13 | Clearly describe the research question, research objectives and, where appropriate, specific hypotheses being tested. |
| Ethical statement | 14 | Provide the name of the ethical review committee or equivalent that has approved the use of animals in this study, and any relevant licence or protocol numbers (if applicable). If ethical approval was not sought or granted, provide a justification. |
| Housing and husbandry | 15 | Provide details of housing and husbandry conditions, including any environmental enrichment. |
| Animal care and monitoring | 16 | a. Describe any interventions or steps taken in the experimental protocols to reduce pain, suffering, and distress. b. Report any expected or unexpected adverse events. c. Describe the humane endpoints established for the study, the signs that were monitored, and the frequency of monitoring. If the study did not have humane endpoints, state this. |
| Interpretation/scientific implications | 17 | a. Interpret the results, taking into account the study objectives and hypotheses, current theory, and other relevant studies in the literature. b. Comment on the study limitations, including potential sources of bias, limitations of the animal model, and imprecision associated with the results. |
| Generalisability/translation | 18 | Comment on whether, and how, the findings of this study are likely to generalise to other species or experimental conditions, including any relevance to human biology (where appropriate). |
| Protocol registration | 19 | Provide a statement indicating whether a protocol (including the research question, key design features, and analysis plan) was prepared before the study, and if and where this protocol was registered. |
| Data access | 20 | Provide a statement describing if and where study data are available. |
| Declaration of interests | 21 | a. Declare any potential conflicts of interest, including financial and nonfinancial. If none exist, this should be stated. b. List all funding sources (including grant identifier) and the role of the funder(s) in the design, analysis, and reporting of the study. |

**11-13.** Our abstract, and background sections contain the recommended content.

**14.** All experiments were approved by the Biomedical Ethical Committee of Peking University (No. LA2022367).

**15.** The experimental animals were raised in iron cages with sufficient space to move around.

**16. a.** Ibuprofen were injected before the operation, and 1000 mL of glucose and sodium chloride were infused intravenously during the operation. After the operation, ibuprofen was intramuscularly injected for 14 days. **b.** CT examinations revealed that the reconstructed condyle in one pig had split at two weeks. In another case, the operative area had been infected and failed to recover after debridement. **c.** The experimental animals excluded due to adverse events were euthanized. The serious adverse events include condylar fracture, necrosis, and infection. The condition and wounds of the animals were observed every day, and some adverse events were detected by CT examinations.

**17-18.** Our discussion sections contain the recommended content.

**19.** We prepared a protocol before the study without registration.

**20.** The data sets used and/or analyzed during the present study are available from the corresponding author upon reasonable request.

**21. a.** The authors declare that they have no competing interests. **b.** The article was supported by the Program for New Clinical Techniques and Therapies of Peking University School and Hospital of Stomatology (grant PKUSSNCT-21G01) and the National Program for Multidisciplinary Cooperative Treatment on Major Diseases (PKUSSNMP-202007).

**Additional Fig. 1** Postoperative body temperature curves of experimental pigs. 1, 2, 3 indicates three pigs included in the outcome analysis.

**Additional Fig. 2** Weight curves of experimental pigs. Pigs was measured preoperatively, and at three months, and six months postoperatively under anesthesia. The animals were fasted for 12 hours before weighing. 1, 2, 3 indicates three pigs included in the outcome analysis.
